# Acute Effects of Blood Transfusion on Insulin Sensitivity and Pancreatic β-Cell Function in Children with β-Thalassemia/Hemoglobin E Disease

**DOI:** 10.4274/jcrpe.4774

**Published:** 2018-02-26

**Authors:** Somboon Wankanit, Ampaiwan Chuansumrit, Preamrudee Poomthavorn, Patcharin Khlairit, Sarunyu Pongratanakul, Pat Mahachoklertwattana

**Affiliations:** 1 Mahidol University Faculty of Medicine, Ramathibodi Hospital, Department of Pediatrics, Divisions of Endocrinology and Metabolism, Bangkok, Thailand; 2 Mahidol University Faculty of Medicine, Ramathibodi Hospital, Department of Pediatrics, Divisions of Hemato-Oncology, Bangkok, Thailand

**Keywords:** Insulin resistance, hemoglobinopathy, iron, hemochromatosis, ferritin

## Abstract

**Objective:**

To assess the acute effects of blood transfusion on insulin sensitivity and pancreatic β-cell function in thalassemia patients.

**Methods:**

Fifty children and adolescents with β-thalassemia/HbE disease were enrolled in a prospective cohort study. Hemoglobin, serum ferritin and oral glucose tolerance test (OGTT) were performed prior to, and one week after blood transfusion. Insulin sensitivity indices [homeostatic model assessment (HOMA) of insulin resistance (HOMA-IR), whole body insulin sensitivity index (WBISI)] and β-cell function indices [HOMA of β-cell function (HOMA-β), insulinogenic index (IGI), and disposition index (DI)] were calculated from glucose and insulin levels obtained during the OGTT.

**Results:**

Following blood transfusion, hemoglobin and serum ferritin increased significantly; 8.5 to 10.1 g/dL (p<0.001) and 1764 to 2160 ng/mL (p<0.001), respectively. β-Cell function indices also increased significantly [median HOMA-β: 74.3 vs. 82.7 (p=0.033); median IGI: 59.6 vs. 79.3 (p=0.003); median DI: 658 vs. 794 (p=0.01)]. However, the insulin sensitivity index (WBISI) tended to decrease and the insulin resistance index (HOMA-IR) tended to increase although this did not reach significance. Multivariate analysis showed that pre-transfusion serum ferritin was the major factor negatively associated with WBISI and positively associated with HOMA-IR, but pre-transfusion hemoglobin had no significant association with insulin sensitivity indices post-transfusion.

**Conclusion:**

This study demonstrated that acute increases in serum ferritin and hemoglobin following blood transfusion in patients with thalassemia might contribute to an increase in insulin secretion and to a trend towards increased insulin resistance.

## What is already known on this topic?

Chronic iron overload in transfusion-dependent thalassemia patients is a cause of insulin resistance and pancreatic β-cell dysfunction. In addition, severe anemia is also associated with insulin resistance. Essential regular blood transfusions will improve the anemic state in severe thalassemia but will also increase iron accumulation.

## 

### What this study adds?

This is the first study that reports the acute effects of blood transfusion on insulin sensitivity and β-cell function. We demonstrated that following blood transfusion in thalassemic patients, acute iron loading accompanied by partial correction of anemia, resulted in a rise in insulin secretion and a trend towards increasing insulin resistance.

## Introduction

Thalassemia is a hereditary hemolytic disease caused by hemoglobinopathy. Among patients with moderate to severe disease, regular blood transfusion is a vital modality of treatment. However, chronic blood transfusions result in an increase in total body iron, thus iron overload, which in turn leads to iron deposition in multiple organs, including the pancreas. In animal studies, intravenous iron loading was shown to lead to pancreatic necrosis by free radical oxygen species through a Fenton reaction (1,2). Glucose dysregulation, secondary to increased insulin resistance and pancreatic b-cell dysfunction, has been widely reported in thalassemia major patients with iron overload (3,4). Furthermore, adverse effects of iron overload on insulin sensitivity and insulin secretion varied, depending on the degree of iron excess and may be reversible after reduction of tissue iron accumulation. In patients with hereditary hemochromatosis, there was a high prevalence of diabetes mellitus and impaired glucose tolerance (IGT) (5). Besides, normalization of serum ferritin by phlebotomy improved insulin secretory capacity (6). In addition, we recently demonstrated that there was a trend towards improvement of insulin sensitivity and b-cell function following six months of iron chelation therapy in adolescents with non-transfusion-dependent thalassemia (7). Phlebotomy in diabetic patients and blood donation in normal individuals were also shown to improve insulin sensitivity, and thus decrease insulin secretion (8,9). 

In addition to increasing the serum iron level, blood transfusions immediately improve the anemic state. Anemia has been shown to be associated with increased insulin resistance in chronic renal failure. Previous studies of chronic renal failure patients treated with multiple episodes of hemodialysis reported that low hematocrit (Hct)  may induce tissue hypoxia and cause insulin resistance. Erythropoietin treatment improves the anemic state and reduces insulin resistance in these patients (10,11,12). 

Blood transfusion has also been shown to acutely raise serum ferritin level while simultaneously improving the anemic state. These effects can be detected within a week following blood transfusion (13,14). However, the acute effects of blood transfusion on insulin sensitivity and b-cell function in patients with thalassemia remain unknown. We therefore hypothesized that iron loading, along with improvement of the anemic state following blood transfusion might have negative effects on insulin sensitivity and might adversely increase b-cell function.

## Methods

This prospective cohort study was conducted at the Department of Pediatrics, Faculty of Medicine, Ramathibodi Hospital, Bangkok, Thailand during the period from April 2015 to March 2016. Children and adolescents aged 5-20 years and diagnosed with b-thalassemia/hemoglobin (Hb) E disease, were enrolled into the study. All patients required regular, packed red cell transfusions every four weeks. Patients with other systemic illness, such as diabetes mellitus, chronic renal disease, cardiomyopathy and those taking medications affecting insulin sensitivity and b-cell function or who had had bone marrow transplantation performed were excluded. Anthropometric data including weight, height, body mass index (BMI) and Tanner’s pubertal stage were recorded. Standard deviation (SD) scores of weight and height were calculated using the National Standard Growth Curve of the Ministry of Public Health, Thailand (15). Duration of the disease and median serum ferritin level during the past 3 years were recorded.

After an overnight fast, an oral glucose tolerance test (OGTT) was performed and blood samples were taken for measurement of basal Hb, Hct and serum ferritin. The patients then proceeded to receive packed red cell transfusions at a standardized dose of 10 mL/kg. One week following the transfusion, all patients underwent a second OGTT as well as measurement of Hb, Hct and serum ferritin. 

Hb was measured using an Abbott Cell Dyne Ruby hematology analyzer (Abbott Diagnostics, Lake Forest, Illinois, USA) within four hours after blood collection. Vitros ferritin assay (Ortho Clinical Diagnostics, Johnson & Johnson, UK) was used for serum ferritin measurement. For glucose and insulin level assessments, Abbott ARCHITECT c16000 Clinical Chemistry Analyzer (Abbott Diagnostics, Lake Forest, Illinois, USA) and IMMULITE chemiluminescent immunoassay (Siemens Medical Solutions Inc., Malvern, Pennsylvania, USA) were used, respectively.

OGTT was performed in the morning, following an eight-hour overnight fast, using 1.75 g glucose/kg body weight (maximum 75 g). Plasma glucose and serum insulin concentrations were determined before and at 30, 60, 90 and 120 minutes following the ingestion of the glucose solution. Each sample was immediately sent to the laboratory right after blood drawing for analytic procedure. The result of the OGTT was interpreted according to American Diabetes Association criteria (16). 

Homeostatic model assessment (HOMA) of insulin resistance  (HOMA-IR) (17), and whole body insulin sensitivity index (WBISI) (18) were calculated to determine insulin sensitivity. For b-cell function, HOMA of b-cell function (HOMA-b) (19), insulinogenic index (IGI) (20) and disposition index (DI) (21) were calculated for assessment of b-cell function. areas under the curve (AUC) of glucose and insulin were calculated using the trapezoidal method. The above-mentioned indices were calculated using the previously published formulas as shown below.

HOMA-IR= [Glucose 0 min (mmol/L) x Insulin 0 min (μIU/mL)] / 22.5

WBISI= 10000 / √[Glucose 0 min (mg/dL) x Insulin 0 min (μIU/mL) x Mean glucose (mg/dL) x Mean insulin (μIU/mL)]

HOMA-β= [20 x Insulin 0 min (μIU/mL)] / [Glucose 0 min (mmol/L)-3.5]

IGI= [Insulin 30 min-Insulin 0 min (pmol/L)] / [Glucose 30 min-Glucose 0 min (mmol/L)]

DI=WBISI x IGI

This study was approved by the Faculty of Medicine Ramathibodi Hospital, Mahidol University (approval number: 04-58-07; date: 22.04.2015). A written informed consent was obtained from all patients and their legal guardians before the enrollment.

### Statistical Analysis

The data were analyzed using IBM SPSS Statistics version 24.0 (SPSS Inc., Illinois, USA). Data are presented as mean ± SD for parametric data and median (interquartile range, IQR) for non-parametric data. Paired-samples t-test and Wilcoxon signed-rank test were performed to compare differences between pre- and post-transfusion parameters for parametric and non-parametric parameters, respectively. A chi-square test was used to compare OGTT results between pre-transfusion and post-transfusion samples. Spearman rank test was used for analysis of correlation among parameters. Linear log regression was used for multivariate analysis. A p value of less than 0.05 was considered statistically significant.

## Results

Fifty children and adolescents with b-thalassemia/HbE disease were enrolled in this study. Clinical characteristics of the patients are shown in Table 1. 64% (32/50) were pubertal and 76% (38/50) had been receiving only one kind of iron chelator, deferiprone. Short stature and underweight were common. None of them was obese (median Z-score of BMI -0.40, IQR -1.35 to 0.09). Only eight patients (16%) had a previous history of splenectomy. Serum ferritin levels during the three years prior to enrollment were high (median 1725, range 362-5740 ng/mL). Acute illness was not observed in our patients during the one-week post-transfusion period. 

Results of OGTT between pre- and post-transfusion were compared (Table 2). Forty-three (86%) had normal glucose tolerance (NGT) at both pre- and post-transfusion assessments. Two patients had IGT at both tests. Two patients with NGT before transfusion developed IGT at post-transfusion. One patient with NGT at pre-transfusion had impaired fasting glucose (IFG) at post-transfusion. Conversely, one patient with IGT and another with IFG at pre-transfusion had normal OGTT result at post-transfusion. There was no significant difference between pre- and post-transfusion groups.

Following blood transfusion, there were significant increases in mean Hb (8.5 to 10.1 g/dL), mean Hct (26.6 to 31.4%) and median serum ferritin level (1764 to 2160 ng/mL) (Table 3). Insulin sensitivity tended to decrease following the transfusion as defined by a reduction in WBISI and an increase in HOMA-IR, but these were not statistically significant. However, b-cell function indices, including HOMA-b, IGI and DI were all significantly increased following transfusion. Additionally, AUC of plasma glucose obtained during the OGTT pre- and post-transfusion was not significantly different. However, AUC of serum insulin at pre-transfusion was significantly lower than that of the post-transfusion point.

Correlation analysis of pre-transfused Hb and serum ferritin with insulin sensitivity and b-cell function indices showed that only pre-transfused ferritin had a negative correlation with percentage change of WBISI (r=-0.32, p=0.031), (data not shown). 

Differences in total body iron and anemic state potentially influence the changes in insulin sensitivity and b-cell function. We therefore performed subgroup analysis according to pre-transfused Hb and pre-transfused serum ferritin (Table 4). Since most of our patients were sub-optimally transfused [mean (SD) pre-transfused Hb 8.5 (1.1) g/dL] and sub-optimally iron-chelated [median (IQR) pre-transfused serum ferritin 1764 (799-2662) ng/mL], Hb of 8.5 g/dL and serum ferritin of 1500 ng/mL were used as the cut off points for subgroup analysis. In the low pre-transfused Hb group (<8.5 g/dL), there were significant increases in all b-cell function indices (HOMA-b, IGI, DI), but no significant changes in insulin sensitivity indices (HOMA-IR, WBISI) following the transfusion. In comparison, no significant changes in either b-cell function or insulin sensitivity indices were observed in the high pre-transfused Hb group (≥8.5 g/dL). In the high pre-transfused serum ferritin group (>1500 ng/mL), there was a significant decrease in insulin sensitivity (decreasing WBISI and increasing HOMA-IR) and significant increases in b-cell function indices (HOMA-b, IGI) following transfusion. In contrast, in the low pre-transfused serum ferritin group, there were no changes of insulin sensitivity indices (WBISI**, **HOMA-IR) and b-cell function indices (HOMA-b, IGI), but slightly and significantly increased DI following the transfusion. Using multivariate analysis, pre-transfused serum ferritin was the only factor associated with changes of HOMA-IR (F=4.080, p=0.049) and WBISI (F=6.799, p=0.012).

## Discussion

Studies in patients with thalassemia and hereditary hemochromatosis who had chronic iron overload showed that glucose dysregulation occurred as a result of insulin resistance followed by b-cell dysfunction (5,22,23,24). Excessive iron causes insulin resistance and subsequently, pancreatic b-cell apoptosis and insulin deficiency (25,26). Most of our patients had NGT at both pre-transfusion and post-transfusion assessments. Their AUC of plasma glucose remained unchanged following the transfusion, while AUC of serum insulin was increased significantly at post-transfusion, reflecting a rise in b-cell function. In fact, in the early phase of glucose dysregulation, change of insulin secretion is reciprocal to that of insulin sensitivity. In parallel with AUC of serum insulin, all b-cell function indices were also elevated following blood transfusion. Meanwhile, there was a trend towards decreasing insulin sensitivity. Therefore, we speculate that acute iron loading, concomitant with partial correction of anemia following a single dose of packed erythrocytes, resulted in a trend towards reduction in insulin sensitivity and thus caused a rise in insulin secretion. 

Among patients with chronic renal failure treated with hemodialysis, moderate to severe anemia was also a risk factor for insulin resistance (10,11,12,27). Partial correction of anemia with erythropoietin was shown to reduce insulin resistance as well as reduce insulin secretion (11,12). In chronic renal failure, erythropoietin treatment improved the anemic state with no change of iron status (11,12). In contrast, blood transfusions in thalassemic patients improve the anemic state, but lead to increased iron load.

Considering the present study, acute iron loading from blood transfusion with an increase in serum ferritin about 400 ng/mL concomitant with an increase in Hb of about 1.5 g/dL caused increased insulin secretion and may have had a detrimental effect on insulin sensitivity. In addition, the acute change in total body iron within only a week may cause increased insulin resistance in chronically transfused patients, particularly in those with relatively high pre-transfused serum ferritin. As shown in the multivariate analysis, pre-transfused serum ferritin was the only factor associated with insulin sensitivity indices.

To the best of our knowledge, acute effects of blood transfusion on insulin sensitivity and b-cell function have not been reported in thalassemia patients. Early changes in insulin sensitivity and b-cell function may help in understanding the sequences of pathophysiology underlying glucose dysregulation in thalassemia patients.

### Study Limitations

There were some limitations in this study. Firstly, the majority of the patients were sub-optimally transfused and sub-optimally iron-chelated. A high iron store may mask the actual effect of correction of anemia on insulin sensitivity and b-cell function. Secondly, since b-thalassemia/HbE disease has a spectrum of severity, that is varied degrees of hemolysis and transfusion requirement, these may be important factors determining insulin sensitivity and b-cell function. Thirdly, a larger sample size is required for the subgroup analysis to distinguish the effects of anemia from iron overload on insulin sensitivity and b-cell function. Understanding the relationship between Hb and glucose homeostasis, independently of iron status would be beneficial in identifying optimal target Hb for maintaining near-normal insulin sensitivity and b-cell function.

## Conclusion

Our study has demonstrated that acute increases in serum ferritin and Hb following blood transfusion in patients with b-thalassemia may contribute to an increase in insulin secretion and thus to a trend towards increased insulin resistance.

## Figures and Tables

**Table 1:**
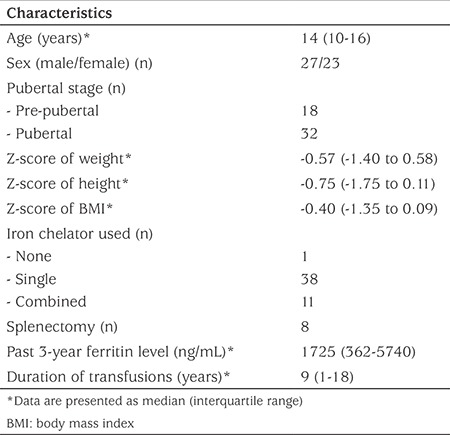
Clinical characteristics of all enrolled patients (n=50)

**Table 2:**
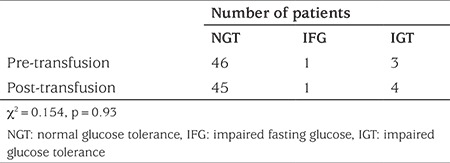
Pre-transfusion and post-transfusion oral glucose tolerance test results of all enrolled patients (n=50)

**Table 3:**
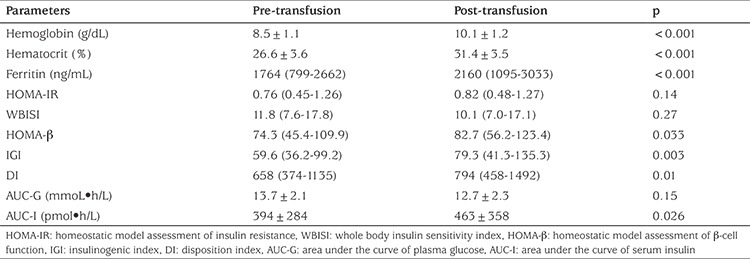
Pre- and post-transfusion values for hemoglobin, hematocrit (mean ± standard deviation), serum ferritin, insulin sensitivity and β-cell function indices (median, interquartile range) and area under the curve of glucose and insulin (mean ± standard deviation) in 50 thalassemic children and adolescents

**Table 4:**
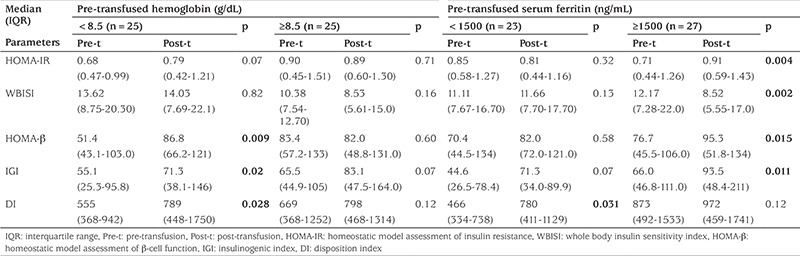
Insulin sensitivity and β-cell function indices at pre- and post-transfusion according to pre-transfused hemoglobin and pre-transfused serum ferritin
